# Content of Iron and Vitamin A in Common Foods Given to Children 12–59 Months Old from North Western Tanzania and Central Uganda

**DOI:** 10.3390/nu11030484

**Published:** 2019-02-26

**Authors:** Beatrice Ekesa, Deborah Nabuuma, Gina Kennedy

**Affiliations:** 1 Bioversity International, Plot I06, Katalima Road, Naguru, Kampala P.O. Box 24384, Uganda; d.nabuuma@cgiar.org; 2 Bioversity International, Via dei Tre Denari, 472/a, 00054 Maccarese, Rome, Italy; g.kennedy@cgiar.org

**Keywords:** complementary foods, iron, Tanzania, Uganda, vitamin A

## Abstract

Improving infant and young child feeding is an effective intervention to improve child growth. A cross-sectional study followed by observation of selected households was used to establish the most popular foods given to children 12–59 months old in Bukoba and Kiboga districts of Tanzania and Uganda, respectively. Six meals were identified: maize-based porridge, steamed-mashed banana served with beans, banana cooked with beans, banana cooked with groundnut sauce, stiff porridge (*Ugali*) served with beans and sardines, and cassava cooked with beans. Raw ingredients were transported to Universität für Bodenkultur, Austria, within 48 h and meals prepared following community validated procedures within 24 h by project team members that involved graduate students from East Africa and Europe. High-performance liquid chromatography (HPLC) analysis and microwave digestion followed by flame atomic absorption spectroscopy were used in establishing provitamin A carotenoids and iron content, respectively. Findings indicated no trace of vitamin A or iron in the maize-based porridge, whereas 2.28 mg/100 g ep (edible portion) and 1.18 mg/100 g ep of iron were recorded in stiff-porridge served with beans and sardines and banana cooked with beans, respectively. Banana-based foods had 23 to 43 vitamin A RAE (retinal activity equivalent) µg/100 g ep. With estimated average requirements of iron and vitamin A for children 1–3 years being 5 mg/day and 275 RAE µg/day, respectively, these foods are poor sources of these nutrients in their current form. Thus, there is a need to explore opportunities for modifying preparation methods and incorporating nutritious and diverse ingredients into the foods prepared for infants and young children in Eastern African countries.

## 1. Introduction

The global strategy on child feeding recommends that after six months of exclusive breastfeeding, infants and young children should receive safe and nutritionally adequate complementary foods. That is, foods that provide sufficient energy, protein, and micronutrients to meet the child’s nutritional requirements [1,2]. This transition period has been marked with increased nutrient requirements, rapid growth, and sadly increased incidence of malnutrition and infection [3]. Improving complementary feeding has been found to be an effective intervention to improve child growth and reduce stunting [1]. However, two billion people worldwide are estimated to have micronutrient deficiencies, particularly iron, vitamin A, zinc, and iodine [4]. The effects of these deficiencies include poor health, mental impairment, low productivity, and death, and are particularly more severe within the first 1000 days of life, from conception to two years of age. These deficiencies are usually a result of, increased needs during certain life stages, inadequate diets, diseases, infections, and/or parasites [5]. Occurrence of more than one micronutrient deficiency is common in low- and middle-income countries due to inadequate diets, especially of vulnerable groups such as children and mothers [6].

The 2016 global hunger index scores for Uganda and Tanzania are 26 and 28, respectively—a severity rated as serious [5]. The score is strongly related to measures of hidden hunger such as indicators of vitamin A deficiency and anemia, and diet quality of children [5]. In Uganda and Tanzania, the levels of vitamin A deficiency are 39% and 43% among children below five years old, respectively [4]. Half of the children under five in Uganda and Tanzania have iron deficiency anemia (58% in Tanzania and 53% in Uganda) [7,8].

Diets of children below five years in sub-Saharan Africa are characterized by starchy staples with limited consumption of animal source foods, fruits, vegetables, or pulses, and are thus an influencing factor towards micronutrient deficiency [7,8,9,10]. This study explores the vitamin A and iron content of popular foods given to children in North Western Tanzania (Bukoba district) and Central Uganda (Kiboga district) in order to be able to ascertain their current contribution to nutrient needs and provide the basis for future research and development interventions focusing on improving nutrient content of diets for young children.

## 2. Materials and Methods

### 2.1. Identification of Common Complementary Dishes

A cross-sectional survey of more than 400 households with preschool children in Bukoba district, North Western Tanzania (*n* = 206) and Kiboga district, Central Uganda (*n* = 219) was carried out to establish the most popular foods given to children aged 12–59 months. Using SPSS Version 16 (SPSS Inc, Chicago, IL, USA) for Windows, the frequency in consumption of each meal/food was identified and out of these foods the three most popular ones were identified, and the most common combination of ingredients used to prepare them were established. Following this, the dishes mentioned by 10% or more of the study sample were purposively designated as the most common foods. After the establishment of these dishes, follow-up home visits that incorporated close observation and in-depth interviews of 11 randomly selected households in each of the two countries/districts were carried out to confirm and obtain the specific preparation procedures of the identified dishes. The preparation methods varied slightly from family to family, but the fundamentals were the same across the different families visited. Local household measures like cups, spoons, and plates/bowls were used to establish quantities and equated to standard measures. Table 1 provides detailed description of the selected most popular dishes including specific ingredients and preparation procedures.

Fresh ingredients for the six popular foods were obtained from the study sites (local markets in Bukoba and Kiboga) by the research team, packaged appropriately, and transported to Vienna (Institute of Food Science, Universität für Bodenkultur BOKU, Austria) as hand luggage with relevant documentation within 48 h of purchase. On arrival at the laboratory, the graduate students from the research team prepared the respective meals as per the procedures verified at household level. Cooked samples of each of the ingredients were also collected and individually analyzed before meal preparation. Both the single ingredients and the dishes/meals were stored at −18 °C to await analysis (not more than 14 days). A portion of each sample (20–40 g) was frozen at −24 °C for 6 h and freeze-dried for 24 h (Freeze Dryer Modulyo, Edwards, Bolton, UK). Dry matter was determined, and the samples were then homogenized (Osterizer, Pulsematic 10, Sunbeam Products, Inc., Boca Raton, FL, USA) and stored at −24 °C until analysis. 

### 2.2. Vitamin A/Carotenoids Analysis

Most of the foods were plant-based or contained negligible animal origin compounds (Table 1); analysis was therefore focused on provitamin A carotenoids (pVACs). Carotenoids are very sensitive to light and oxygen. Exposure to light leads to *trans-cis* isomerization and destruction of pVACs. Therefore, carotenoid analysis was carried out as fast as possible under subdued light and flasks wrapped with aluminum foil [11]. 

Analysis was carried out in triplicate with the freeze-dried samples dissolved in *trans*-β-apo-8’-carotenal as internal standard and petroleum ether. A rotary evaporator was used to vaporize the petroleum ether. To eliminate existing lipids, the remaining extract was dissolved in acetone and frozen at −24 °C for 3–4 h. The frozen acetone was then added, and the liquid sample was filtered to separate fat from the carotenoids [11]. The filtered samples were stored at –24 °C under argon awaiting analysis. Following this, a linear gradient elution was used for the HPLC analysis. For quantification of the carotenoids, a calibration curve was established. For this, an internal standard using *trans*-β-apo-8’-carotenal and an external standard β-carotene were prepared at different concentrations [12].

According to their characteristic absorption spectra and retention times (compared to the added standards), carotenoids of each sample were identified and quantified [12]. Chromquest 5.0 Software (Termo Fisher Scientific, Inc., Waltham, MA, USA) was used for the interpretation of the chromatograms. Values of peak areas were used to calculate the carotenoid contents. The areas under the curve ratios between *trans*-β-apo-8’-carotenal as internal standard and the compounds were used for the determination of concentrations [13]. Calculations were carried out using Microsoft Office Excel 2007 (Microsoft, Redmond, WA, USA).

### 2.3. Iron Analysis

For the determination of the samples’ iron content, a microwave digestion was carried out followed by a flame atomic absorption spectroscopy (FAAS). To analyze the iron concentration in the AAS, the sample solution was transformed into aerosols and transported to the flame, which vaporizes and atomizes the sample. This step leads to a reduced intensity of the light (by absorption of a defined quantity of energy) coming from a hollow-cathode lamp. A following detector measures how much of the incoming light was absorbed. The difference between the radiation without sample and including sample (absorbance) was used to calculate the iron concentration. Freeze-dried samples of all dishes were used for the analysis.

The AAS measurement started with the analysis of the blank value, followed by the standards and the samples. On the basis of the calibration curve and the specific absorption of the different samples, the AAS calculates the iron concentration of each sample. Concentration values were used for the calculation of the total iron content.

### 2.4. Statistical Analysis

Using SPSS software version 16, the data generated from each sample following nutrient analysis was subjected to statistical analysis using two-way analysis of variance (two-way ANOVA) tests and the difference in mean was compared using the Duncan’s new Multiple Range test (*p* ≤ 0.05) (Duncan, 1955). This was done to test for differences of nutrient contents among the different foods and individual ingredients. The average value of the triplicates was used in our results section and compared to estimated average requirements (EAR). EAR for iron is 3 mg/day for infants aged 1–3 years and 4.1 mg/day for children aged 4–8 years; EAR for vitamin A is 210 RAE µg/day for infants aged 1–3 years and 275 µg/day for children aged 4–8 years [14].

## 3. Results and Discussion

### 3.1. Common Foods and Cooking Procedures

Four hundred and twenty-five households with children less than five years old were interviewed in Tanzania and Uganda. Following a question where they were requested to mention and describe the three most popular foods given to children between 12–59 months old in their community, a total of 482 versions of foods were mentioned. While this seems to be a large number, often the variance between prepared foods was based on only one or two ingredients. For instance, for porridge there were more than 10 versions mentioned because of the different flours used like maize, millet, cassava, soya, and the different flour combinations where two or more were used in one recipe. Communities combine flours due to the known nutrition complementarities that different flours bring and to enhance the consistency of the porridge. In addition to the difference in flours, there were many ingredient combinations, such as flour with milk, sugar, and water; flour with sugar and water; flour with just water; and flour with milk and water. For these reasons only, foods mentioned by more than 10% of the interviewed households in each of the two country sites were considered popular, thus resulting in six unique foods as described in Table 1. Based on the average household size in Uganda and Tanzania of 4–6 members (15, 8), the quantity and proportion of ingredients used were based on a five-serving portion.

Table 1 shows that the most common foods for both countries were based on maize, banana or cassave, with the main additional ingredients being beans and groundnuts. One of the common foods in Tanzania contained sardines/small fish (dagaa), while none of the top common foods in Uganda contained any animal source food as a main ingredient. This can be supported by the proximity of the population in Tanzania to the western shore of Lake Victoria, where fishing is a main economic activity and small fish (dagaa) are the cheapest type of fish [15]. 

Although some of the recipes included vegetables such as tomatoes and onions, the proportion of these vegetables compared to the volume of the whole meal was too low to make any substantial contribution to the nutrient quality of the overall meal. It is also important to note that none of the six recipes include green leafy vegetables. This supports previous studies that have documented that most of the foods given to children below five years are starchy based with little or no animal source foods, vegetables, and fruits [16]. Regular consumption of these common foods has resulted in a very low dietary diversification score, as starchy foods provide almost three quarters of the total energy supply, despite the wide variety of food produced in both Uganda and Tanzania [17]. 

In addition, the cooking methods indicate that most of the common meals are simply boiled with little or no addition of oil, and the cooking durations described can be considered very long, likely contributing to the loss of nutrients and also inhibiting nutrient absorption. Exposure of the food to extreme heat during prolonged cooking results in oxidative destruction of carotenoids while the presence of dietary fat improves absorption of the carotenoids [18]. The procedures do not indicate any effort to either minimize cooking time or enhance nutrient availability; this is seen in the case where dry beans were used but soaking was not done. The phytic acid and polyphenol content that is reduced during soaking and the act of throwing away the water enhances the bioavailability of iron in the beans [19,20]. It is important to note that the preparation methods of the foods in this study are like those for the rest of the household. Results from a separate study from the same sites show that less than 50% of households prepare special meals for the children [21]. Other studies also report that most children consume foods from family meals [3,10]. Efforts to improve the quality of complementary foods should ensure that the technologies are suitable for the household and are accompanied with nutrition education [22,23].

### 3.2. Iron and Vitamin A Content in the Specific Ingredients and the Recipes/Dishes

To fully understand the nutrient composition of the foods, an analysis of the individual ingredients was carried out. Table 2 shows the content of iron and provitamin A carotenoids in the ingredients. Of all the food items/ingredients, only ‘Nshakala’ a cooking banana local to East Africa seem to have some pVACs (>600 µg/100 g ep); this can be explained by the great worldwide diversity in banana varieties (>1000 varieties) and the fact that the screening of just 300 varieties indicated a high degree of variability in the pVAC content levels of bananas, ranging from 6.85 to 2365 RAE µg/100 g ep [24]. Based on bananas being a major part of the diets for infants and young children from both Uganda and Tanzania, as observed here, appropriate utilization of the diversity in bananas could provide a feasible entry point of enhancing the vitamin A intake among infants and young children. 

Regarding iron, the sardines/small fish (mukene/dagaa) and kidney beans contained considerable amounts of iron (>1.5 mg/100 g ep (edible portion)). Food items such as maize, cassava, and groundnuts did not have any substantial amounts of either pVACs or iron.

The findings in the recipes shown in Table 3 indicate that the inclusion of beans in most of the dishes provided considerable amounts of iron and was the only source of this nutrient in the maize-based dishes. The dish with the highest iron content was stiff porridge served with beans and sardines/small fish (2.3 mg/100 g ep), followed by the banana boiled with beans (1.2 mg/100 g ep). This is in line with the content of individual ingredients that indicates that beans and sardines/small fish have the highest content of iron (Table 2). In addition, the foods with neither beans nor sardines had the lowest iron content (Table 3). Therefore, with the already noted wide range of genetic variability of iron content in beans ranging from 3.4 to 8.9 mg/100 g ep [25], the bean variety being used in the children’s foods is important.

Similarly, foods containing banana, the ingredient with the highest pVAC value, contained the highest total pVAC: banana boiled with beans (722 µg/100 g ep), followed by banana boiled with groundnut sauce (505 µg/100 g ep), while cassava boiled with beans had the least pVAC content (33 µg/100 g ep). 

### 3.3. Contribution of the Common Recipes to Iron and Vitamin A Estimated Average Requirements (EAR) for Children Aged 1–3 and 4–8 Years

All the six foods have a higher β-carotene content than α-carotene, thus implying good provitamin A activity. β-carotenoids have a higher rate of absorption and biological activity of provitamin A compared to α-carotenoids [18,26]. The foods that provided the highest content of the two micronutrients and thus make the highest contributions to the iron and vitamin A EARs of children aged 1–8 years were banana boiled with beans; steamed banana and groundnut sauce with sardines; and steamed banana and groundnut sauce with sardines/small fish (Table 4). Having these three foods is a compromise because even though stiff porridge and beans had the highest iron content, it had the second least pVAC content. On the other hand, bananas boiled with groundnut sauce, which had a high content of pVAC, also had the lowest iron content among the six foods. The difference in nutrient contributions by the different foods highlights the need for dietary diversity—that is, consumption of different food groups, as well as consumption of different foods within a food group. Together, the quality of the diet is improved and so is the micronutrient adequacy [5,27].

Other aspects that can improve the micronutrient quality of these common foods with regard to iron include use of iron-rich bean varieties and/or biofortified beans. There are wild bean varieties that have been noted to have high iron content, while biofortified beans have been bred to contain up to 100 mg/g of iron [19]. The promotion of these beans needs to be coupled with skills on improving the bioavailability of iron in the diet, such as soaking, germination, and or dehulling. In addition, inclusion of more animal source foods, such as meat, eggs, and milk, and/or fruits or vegetables rich in ascorbic acid [19,28] would play a major role in enhancing the quality of diets given to preschool children in Tanzania and Uganda.

For the pVAC content, the addition of cooking oil to the diets, and the disruption of the food matrix through cooking and processing, would help improve the bioavailability. Emphasis, however, needs to be made on prolonged exposure to heat because it increases oxidation of the carotenoids, resulting in the creation of isomers, thus reducing the bioavailability [29,30].

Furthermore, the addition of green leafy vegetables to these complementary dishes would also boost both the iron and pVAC content. Green leafy vegetables, many of which are indigenous to the two study sites, are rich in vitamins, minerals, and antioxidants. Levels of iron of 2–13 mg/100 g fw have been reported as well as pVAC contents as high as 1200 µg/100 g fw. In addition, they have a good content of ascorbic acid that improves the absorption of iron with levels of 2–198 mg/100 g fw [31,32]. 

However, it should be noted that the ability to include diverse ingredients within a household diet is influenced by food prices, culture and preferences, geographical environment and seasons, as well as poverty. Food prices, for example, have been found to affect dietary diversity, in that an increase in food prices increases the consumption of staple foods and reduces foods rich in micronutrients, like animal source foods, fruits, and vegetables [5]. 

Therefore, aspects related to enhancing the quality of complementary diets cannot be communicated in isolation. There is a need for accurate information and skilled support from the family, community, and health care system, and appropriate education on improved use of locally available foods is required [1,2]. At times, it is not the lack food but inadequate knowledge on appropriate, locally available foods and poor feeding practices that result in hidden hunger [2]. Therefore, the design and implementation of any research and development interventions targeting enhanced infant and young child feeding needs to be comprehensive, evidence-based, and suited to the local context and scale in order to yield success [1].

## 4. Conclusions

No trace of either vitamin A or iron was observed in the maize-based porridge, which is a common weaning and complementary dish. Some iron (2.28 mg/100 g fw and 1.18 mg/100 g fw) was observed in the stiff-porridge served with beans and small fish and boiled banana served with red kidney beans, respectively. The banana-based foods showed some content of vitamin A ranging from 23 to 43 RAE µg/100 g fw. Considering the estimated average requirements of iron and vitamin A for children being 4.1 mg/d and 210–275 RAE µg/day, respectively, and the low levels of continued breastfeeding after 12 months (47.7%) and after 24 months (19.5%) reported in the target communities [33], the foods consumed by children aged 12–59 months in North Western Tanzania and Central Uganda are poor sources of vitamin A and iron in their current form. There is therefore a need to explore the available opportunities around modifying the preparation methods and incorporating more nutritious and diverse ingredients for better nutrition content, while keeping in mind the resources available to the community, their tastes and preferences, and the cost of the eventual diets.

## Figures and Tables

**Table 1:** Description of the recipes of the six most popular foods given to children aged 12–59 months in North Western Tanzania and Central Uganda.

Food Number	Food/Meal Description	Ingredients	Preparation and Cooking Procedures	Comment
1	Porridge from refined maize flour (Uganda and Tanzania)	200 g of white refined maize flour; 2 L of water; sugar to taste (50 g)	Put to boil 1.5 L of water. As it comes to a boil, add 0.5 L of water to the 200 g of flour. Mix well to a smooth light paste. Reduce the heat on the boiling water and add the flour-water paste. Stir thoroughly to avoid forming lumps. Let the mixture come to a boil, reduce the heat, and simmer for 10 min with occasional stirring. Do not cover the pan as the porridge boils. When ready, remove the pan from heat, add sugar, stir, and serve.	More than one serving is prepared and stored either in a thermos flask or plastic jug
2	Steamed mashed banana (matooke) served with groundnut sauce (dried sardines—optional) (Uganda)	20 medium bananas; 500 g groundnut flour; 1 medium-sized onion; 2 medium-sized tomatoes; 50 g dried sardines/small fish (mukene/dagaa)—optional; 4 L of water; salt; 4 banana leaves, 2 fibers and some stems (from banana leaf or bunch)	*Steamed banana* Prepare the pan for steaming by placing the stems in the base of the pan. Add the water without completely submerging the stems. Place the cleaned, peeled bananas onto one banana leaf that is folded width-wise. Wrap the neatly arranged bananas and secure the leaf with the banana fibers. Place the wrapped bananas in the saucepan on top of the stems and over evenly with the rest of the leaves, tuck them into the pan. Place the pan on heat to boil. Cook for about 1 h. Thereafter, remove from the fire, remove the covering banana leaves. Gently mash/press the bananas that are inside the first leaf using the palm of your hand. Continue until evenness is felt. Ensure there is sufficient water in the pan, refill if necessary without submerging the banana stems—the water should not touch the food. Place the wrapped and mashed banana back on top of the stems, cover with the other leaves, and return to heat for a minimum of 30 min or until ready to serve (the bananas can keep warm and ready for longer when on low heat). *Groundnut sauce* Evenly chop the onions and tomatoes and put aside. Gradually add 1 L of water to the groundnut flour, mix thoroughly until smooth. Place mixture on low heat, and continuously stir. Do not cover the pan. When the mixture starts to boil, add the chopped onions, tomatoes, and the salt, and stir for one minute. Let the sauce simmer for 15 min. Cook the sauce until the sauce slightly darkens in color and a little oil is visible on the surface. Serve the sauce with the steamed banana while hot.	For the addition of sardines: Lightly fry the onions in the cooking oil, add the tomatoes and sardines. Cook for 5 min. Add the fried sardines to the groundnut sauce at the point where onions and tomatoes would have been added. Stir gently and cook the sauce on low heat for a further 15 min.
3	Banana boiled with groundnut sauce (katogo) (Uganda)	20 medium bananas; 300 g groundnut paste; 2 medium-sized onions; 2 medium-sized tomatoes; salt to taste; 2 L of water	Place the cleaned and peeled bananas in a pan. Add the water until almost all fingers are submerged (1.5 L). Place covered pan on heat and boil for about 15–20 min. In the meantime, using a separate pan, add 0.5 L of water to the groundnut paste and mix to a smooth texture. Evenly chop the onions and tomatoes. After boiling the bananas for 20 min, add the groundnut mixture and allow to boil for 10 min. Add the onions, tomatoes, and salt, and lightly stir to mix ingredients. Leave to cook for another 10 min.	The groundnut paste is from lightly roasted groundnuts.
4	Banana boiled with beans (katogo) (fried sardines—optional) (Tanzania)	10 medium bananas; 250 g dry kidney beans; 250 g sardines (mukene/dagaa); 1 medium-sized onion; 2 medium-sized tomatoes; 1 tablespoon cooking oil; salt to taste; water	Place clean, sorted beans in pan and boil in water for 1.5 h until partially cooked. In a separate pan, place the cleaned, peeled bananas. Add the beans with their water, salt, to the bananas and more water to cover the food, if needed. Cover the pan and boil for 30 min. As the mixture boils, lightly fry the onions in the cooking oil, add the tomatoes, and cook for 3 min. Add the fried onion and tomatoes to the bean-banana mixture; stir gently and cook for a further 15 min.	For the addition of sardines: Lightly fry the onions in the cooking oil, add the tomatoes and sardines. Cook for 5 min. Add the fried sardines to the bean-banana mixture; stir gently and cook for a further 15 min.
5	Stiff maize porridge (Ugali), served with beans and sardines as relish (optional) (Tanzania)	500 g of white refined maize flour; 250 g dry kidney beans; 100 g sardines (mukene/dagaa); 1 medium-sized onion; 2 medium-sized tomatoes; 2 tablespoons cooking oil; salt to taste; water	*Bean sauce* Boil the clean, sorted beans in water and salt until soft (2 h). When served with beans, boil beans until soft; remove water and mash; heat oil in a sauce pan; add onions, let them sauté, then add tomatoes and cook until soft; add in the mashed beans, stir, add in water, and keep stirring; cover and let cook for about 5 min; the relish is ready. The sardines should be cooked as in recipe 4. *Ugali* Bring to boil 1.5 L of water. Remove about 100 mL and set aside. Gradually add the maize flour to the boiling water while continuously mixing until mixture is even and stiff. If the mixture is too stiff and not yet evenly mixed, add a little of the hot water and continue mixing. When the desired consistency is reached, reduce to low heat, cover the pan, and cook for 3 min. Open lid, mix the mixture thoroughly again for about 2 min. Cover the pan and cook for another 3 min.	
6	Cassava boiled with beans (katogo) (Tanzania)	3 medium cassava roots; 125 g dry kidney beans; 3 L water; salt to taste	Place clean, sorted beans in pan and boil in water for 1.5 h until partially cooked. In a separate pan, place the cleaned, peeled, and chopped cassava. Add the beans with their water to the cassava, salt, and more water to cover the food, if needed. Cover with a lid and cook for 30–45 min, occasionally stirring until the cassava is soft and ready.	

**Table 2:** Iron and provitamin A carotenoid contents (pVACs) of both raw and cooked food ingredients used in preparing the common foods.

Food Item Description	Iron (mg/100 g ep)	Provitamin A Carotenoids (µg/100 g ep)			
		All *trans* α-Carotene	All *trans* β-Carotene	*Cis* β-Carotene	Total pVACs
Boiled cooking bananas (Uganda)	0.21 ± 1.031	361 ± 9.81	310 ± 7.47	58.2 ± 3.08	729.2
Boiled cooking banana (Tanzania)	0.21 ± 1.281	575 ± 428.6	574.3 ± 346	162 ± 99.9	1219.5
Plain matooke (steamed-mashed banana)	0.20 ± 0.022	273 ± 8.16	299 ± 12.3	70.5 ± 1.75	642.5
Boiled cassava	0.24 ± 1.255	n.d.	43.1 ± 186	11.90 ± 50.2	55
Plain maize porridge	n.d.	n.d.	n.d.	n.d.	n.d.
Stiff-maize porridge (ugali/posho)	0.63 ± 0.868	n.d.	n.d.	n.d.	n.d.
Groundnut powder sauce	0.93 ± 0.013	n.d.	n.d.	n.d.	n.d.
Groundnut powder sauce cooked with tomato, onion	0.95 ± 0.040	n.d.	47.2 ± 3.97	7.83 ± 0.84	55.03
Groundnut powder sauce cooked with sardines (mukene/dagaa), tomato, onion	1.08 ± 0.002	n.d.	47.2 ± 1.86	10 ± 0.45	57.2
Un-cooked sardines/small fish (mukene/dagaa)	6.33 ± 0.147	n.d.	n.d.	n.d.	n.d.
Steamed sardines (mukene/dagaa)	2.83 ± 1.338	n.d.	n.d.	n.d.	n.d.
Stewed sardines (sunflower oil, onion, and tomato)	2.54 ± 1.048	n.d.	263.0 ± 34	63.13 ± 1	326.13
Red kidney beans	2.29 ± 0.796	n.d.	n.d.	n.d.	n.d.

Values are means of three independent determinations; n.d. = not detected; 100 g ep refers to 100 g edible portion.

**Table 3:** Iron and provitamin A carotenoids content of the six common foods given to children aged 12–59 months in Northwestern Tanzania and Central Uganda.

Name and Description of Common Foods	Iron (mg/100 g ep)	Provitamin A Carotenoids (µg/100 g ep)				t-BCE	RAE
		All *trans* α-Carotene	All *trans* β-Carotene	*Cis* β-Carotene	Total pVACs		
1. Porridge from refined maize flour and water	n.d.	n.d.	n.d.	n.d.	-	-	-
2a. Steamed mashed banana, sardines, groundnut sauce, onions, and tomatoes	0.46 ± 0.050	153±2.13	180 ± 1.52	44.5 ± 1.26	377.50	279.64	23.23
2b. Steamed mashed banana with groundnut sauce	0.43 ± 0.035	154 ± 5.79	196 ± 7.73	48.6 ± 2.18	398.60	298.27	24.78
3. Banana boiled with groundnut sauce, tomatoes, and onions (katogo)	0.31 ± 0.018	202 ± 6.56	240 ± 5.69	63.2 ± 1.86	505.20	373.86	31.05
4. Banana boiled with beans	1.18 ± 0.314	303.7 ± 157.2	326.3 ± 98	92.0 ± 29.9	722.00	525.99	43.68
5. Stiff maize porridge with beans and stewed sardines (dagaa)	2.28 ± 0.791	n.d.	140.1 ± 89	36.43 ± 25.7	176.53	158.94	13.18
6. Cassava boiled with beans	0.89 ± 0.607	n.d.	26.1 ± 2.03	7.27 ± 54.9	33.37	29.88	2.48

The values are means of three determinations ± standard deviation; n.d. = not detected; t-BC = *trans* betacarotene; c-BC = *cis* betacarotene; t-BCE = total batacarotene equivalent; t-BCE = t-BC + c-BC × 0.52 + t-AC × 0.5 in µg/100 g of edible portion; RAE (retinal activity equivalent) = t-BC/12 + c-BC/24 + t-AC/24 in µg/100 g of edible portion. pVACs: provitamin A carotenoids.

**Table 4:** Contribution of the common foods to iron and vitamin A estimated average requirements (EAR) for children aged 1–3 and 4–8 years based on consumption of 100 g edible portion.

Common Foods	Iron Content in the Foods			Vitamin A Content in the Foods		
	mg/100 g	%EAR Child Aged 1–3 Years	%EAR Child Aged 4–8 Years	µg/100 g	%EAR Child Aged 1–3 Years	%EAR Child Aged 4–8 Years
1. Porridge from refined maize flour and water	n.d.	-	-	n.d.	-	-
2a. Steamed mashed banana, sardines, groundnut sauce, onions, and tomatoes	0.46	15.33	11.22	23.23	11.06	8.45
2b. Steamed mashed banana with groundnut sauce	0.43	14.33	10.49	24.78	11.80	9.01
3. Banana boiled with groundnut sauce, tomatoes and onions (Katogo)	0.31	10.33	7.56	31.05	14.79	11.29
4. Banana boiled with beans	1.18	39.33	28.78	43.68	20.80	15.88
5. Stiff porridge with beans and stewed sardines	2.28	76.00	55.61	13.18	6.28	4.79
6. Cassava boiled with beans	0.89	29.67	21.71	2.48	1.18	0.90

EAR for iron is 3 mg/day for children aged 1–3 years and 4.1 mg/day for children aged 4–8 years; EAR for vitamin A is 210 RAE µg/day for children aged 1–3 years and 275 µg/day for children aged 4–8 years [14]; n.d. = not detected.
